# Dementia Screening Intention Among American Indians: The Role of Cultural Orientation and Self-Efficacy

**DOI:** 10.1093/geroni/igaf122.2488

**Published:** 2025-12-31

**Authors:** Heehyul Moon, Yeon-Shim Lee, Soonhee Roh

**Affiliations:** University of Louisville, Louisville, Kentucky, United States; San Francisco State University, San Francisco, California, United States; University of South Dakota, Sioux Falls, South Dakota, United States

## Abstract

Up to one-third of Native American older adults may develop Alzheimer’s Disease and Related Dementias (ADRD). With increasing life expectancy, the number is projected to increase nearly four-fold by 2060. Despite the benefits of early diagnosis, screening rates remain low due to barriers such as cultural beliefs and limited health literacy. This study examined the associations between cultural orientation and dementia screening intention among AI adults in the Yankton Sioux Tribe (YST), with self-efficacy as a potential mediator. A cross-sectional survey was conducted among YST adults (N = 248) recruited through community events (e.g., powwows) using convenience sampling in September 2024. Given low screening rates, this study applied the Theory of Reasoned Action, using screening intention as a proxy for actual behavior. Participants had a mean age of 45 years (range: 18–81); 55% were female, and 65% had a bachelor’s degree or less. Regression analyses revealed that stronger identification with tribal traditions significantly predicted both higher self-efficacy and greater screening intention. Self-efficacy also significantly predicted screening intention. After accounting for self-efficacy, the direct effect of cultural identity on screening intention was reduced but remained significant, indicating partial mediation. These findings suggest that cultural identity enhances screening intention in part by increasing confidence in seeking screening. Findings underscore the need for culturally responsive, self-efficacy-enhancing interventions to improve dementia screening uptake in AI communities. Future research should explore additional psychosocial factors influencing screening behaviors to develop tailored interventions that promote early diagnosis and better health outcomes.

